# Optimization of Mixed Fermentation Conditions of Dietary Fiber from Soybean Residue and the Effect on Structure, Properties and Potential Biological Activity of Dietary Fiber from Soybean Residue

**DOI:** 10.3390/molecules28031322

**Published:** 2023-01-30

**Authors:** Xifei Xu, Xuejing Zhang, Mubai Sun, Da Li, Mei Hua, Xinyu Miao, Ying Su, Yanping Chi, Jinghui Wang, Honghong Niu

**Affiliations:** 1 Institute of Agro-Product Process, Jilin Academy of Agricultural Science, Changchun 133000, China; 2 Department of Food Science and Engineering, College of Agriculture, Yanbian University, Yanji 133002, China; 3 Department of Microbiology, College of Life Sciences, Jilin normal university, Siping 136000, China

**Keywords:** soybean residue, mixed fermentation, modification, dietary fiber

## Abstract

Soybean residue is a by-product of soybean product production that is wasted unreasonably at present. Accomplishing the efficient utilization of soybean residue can save resources. A composite microbial system was constructed using lactic acid bacteria (LAB) and *Saccharomyces cerevisiae* (SC), and modified soybean residue was prepared by solid fermentation. In order to explore the value of modified soybean residue as a food raw material, its physical and chemical properties, adsorption properties, and antioxidant properties were studied. The results showed that the soluble dietary fiber (SDF) yield of mixed fermentation (MF) increased significantly. Both groups of soybean residues had representative polysaccharide infrared absorption peaks, and MF showed a looser structure and lower crystallinity. In terms of the adsorption capacity index, MF also has a higher adsorption capacity for water molecules, oil molecules, and cholesterol molecules. In addition, the in vitro antioxidant capacity of MF was also significantly higher than that of unfermented soybean residue (UF). In conclusion, our study shows that mixed fermentation could increase SDF content and improve the functional properties of soybean residue. Modified soybean residue prepared by mixed fermentation is the ideal food raw material.

## 1. Introduction

Soybean residues are a by-product produced in the process of making soybean products such as tofu and soybean milk which are rich in dietary fiber, polysaccharides, proteins, peptides, and other bioactive substances. Therefore, soybean residues can be used by a variety of microorganisms, improving the added value of soybean residues [1]. However, most soybean residue is currently processed into animal feed, fertilizer, or is discarded directly, resulting in a significant waste of resources [2]. Therefore, it is urgent to transform soybean residue into value-added products through further processing, thereby improving the added value of the soybean residue and accomplishing the high-quality utilization of its organisms.

Dietary fiber (DF) is an important part of maintaining a healthy human diet. According to its solubility, it can be divided into soluble dietary fiber (SDF) and insoluble dietary fiber (IDF). Most plant foods contain a mixture of these two fibers [3]. DF has potential biological activity due to its structural characteristics. Dong et al. [4] found that DF has antioxidant activity. DF has a cholesterol-lowering function [5]. Its antioxidant activity and adsorption capacity were also increased as the proportion of SDF in DF increased, but SDF content was low in natural plant DF. In conclusion, it is very important that we find an effective modification method to improve the content of SDF.

The modification of soybean residue by microbial fermentation is one of the most commonly used modification methods. The microorganisms commonly used are lactic acid bacteria (LAB), yeast, and mixed fermentation; after fermentation, the quality of soybean residue processing was significantly improved [6]. LAB and yeast grow in similar environments, and synergies between microbes may contribute to boosting growth and improving the effect of fermentation [7]. The *Lactobacillus rhamnosus GG* fermentation method improves the water-swelling force and cholesterol adsorption capacity of the dietary fiber in bamboo shoots, as shown by Zhao et al. [8]. Ni-kinmaa [9] discovered that *Lactococcus lactis* can effectively improve rye bran by increasing SDF and soluble protein content while decreasing IDF content. Hassaan [10] used sunflower meal fermented with *Saccharomyces cerevisiae* (SC), and found that the fiber content was reduced while the nutritional value increased. The levels of cholesterol, triglycerides, and high-density lipoprotein in Nile tilapia fed 75% selenite fermented sunflower meal were lower than those in the control group [10]. Furthermore, compared with the fermentation treatment of a single strain, the mixed treatments of two or more strains showed a synergistic effect and overcame their original defects. The mixed fermentation of LAB and SC has been applied to the fermentation of gluten and steamed bread [11,12,13]. Si et al. [14] showed that mixed *Cochliobolus kusanoi* and *Aspergillus puulaauensis* fermentation significantly increased tea residue SDF compared with single bacteria fermentation, and showed a looser structure, lower crystallinity, and higher adsorption capacity in water, oil, and cholesterol molecules. Therefore, mixed fermentation is an effective method to increase the SDF content in soybean residue and improve its activity related to DF.

The aim of this study was to explore the optimal conditions for the mixed fermentation of LAB and SC to improve the SDF in soybean residue. Meanwhile, the effects of mixed fermentation treatment on the structural characteristics, physicochemical properties and potential biological activity of soybean residue were further evaluated. This study could provide new ideas on the modification and preparation of soybean residues.

## 2. Results

### 2.1. Optimization of Fermentation Conditions

Taking SDF yield as the response value, a response surface methodology (RSM) combined with a single-factor test was used to optimize fermentation conditions. The specific factors and levels are shown in Table 1.

Table 2 illustrates the relationship between SDF yield and experimental factors using the quadratic regression equation:

Y = 3.58 − 0.19A − 0.034 × B + 0.020 × C + 0.009983 × D + 0.030 × A × B − 0.022 × A × C − 0.005525 × A × D − 0.039 × B × C + 0.098 × B × D + 0.16 × C × D − 0.34 × A^2^ − 0.35 × B^2^ − 0.18 × C^2^ − 0.68 × D^2^ (1)

among which Y is the SDF yield (g/100 g); A is the water content (%); B is the inoculation proportion (L:S); C is the inoculation amount (%); and D is the fermentation time (h).

The results of the ANOVA used to assess the effect of the quadratic polynomial fitting are shown in Table 3. The results showed that the determination coefficient (R-squared) of the quadratic polynomial was 0.9908, indicating that 99.08% of the total change could be explained by the regression model. When the *p* value lacking the fit was not significant and the *p* value of the regression model was significant, the model showed a good predictive effect. The coefficient of variation (C.V.%) of the regression model was 1.76% < 5%, and the Adeq precision was 32.243 > 4, meaning the model has quite good reproducibility and reliability. The results showed that five quadratic terms (CD, A^2^, B^2^, C^2^, and D^2^) and factor A significantly influenced the SDF yield (*p* < 0.001), while factor B and quadratic term BD had significant effects (*p* < 0.05).

**Figure 1:**
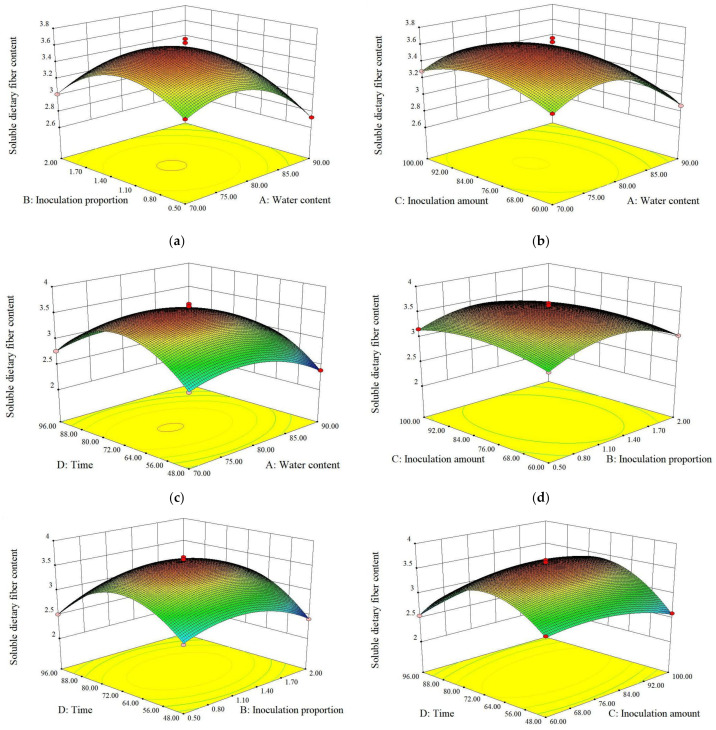
Response surface diagram. Effect of water content and inoculation proportion on SDF yield (**a**); influence of water content and inoculation amount on SDF yield (**b**); effect of water content and fermentation time on SDF yield (**c**); effect of inoculation proportion and inoculation amount on SDF yield (**d**); effect of inoculation proportion and fermentation time on SDF yield (**e**); effect of inoculation amount and fermentation time on SDF yield (**f**).

As shown in Figure 1, the response surface of inoculation amount and fermentation time had the highest inclination, that is, the steepest slope, indicating that the interaction between the two had the most significant effect on SDF yield. The optimal fermentation conditions obtained by the regression model were a water content of 77.22%; an inoculation proportion (L:S) of 6:5; an inoculation amount of 81.67%; and a fermentation time of 72.32 h. The results of the verification experiment showed that the true values agreed with the predicted values of the model, which further confirmed the reliability of the regression model. Therefore, this model can be used to predict the effect of different fermentation conditions on SDF yield.

### 2.2. Structural Properties of Soybean Residue

#### 2.2.1. Scanning Electron Microscope Determination

**Figure 2:**
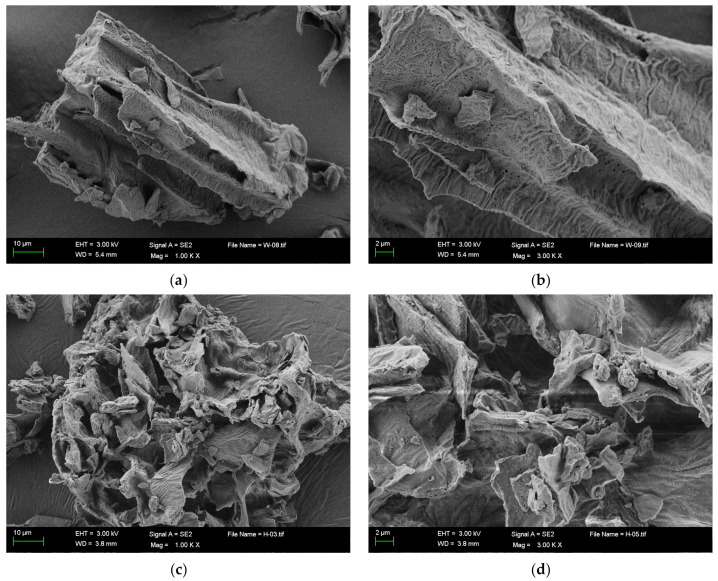
SEM images of UF and MF. (**a**) SEM images of UF × 1000; (**b**) ×3000; (**c**) SEM images of MF ×1000; (**d**) ×3000. UF, unfermented soybean residue; MF, mixed fermented soybean residue.

Scanning electron microscope (SEM) images of the surface of the modified soybean residue samples are shown in Figure 2. The unfermented group (UF) soybean residue had a dense and orderly structure with an obvious intact block and a tight center of particles without holes. The structure of the mixed fermentation treated soybean residue (MF) became very loose and uneven, with many holes and folds and an irregular surface, which laid a solid foundation for improving the physicochemical properties of the soybean residues.

#### 2.2.2. Fourier Transform Infrared Spectroscopy Assay

**Figure 3:**
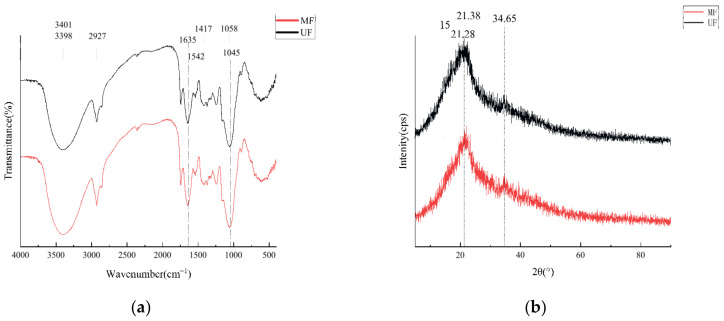
FT-IR (**a**) and XRD (**b**) of mixed fermentation-modified soybean residue. UF, unfermented soybean residue; MF, mixed fermented soybean residue.

Fourier transform infrared spectroscopy (FT-IR) analysis reflected the changes of the functional groups in the soybean residue samples after the mixed fermentation, and the results are shown in Figure 3a. For MF, at 3401, 3398, 2927, 1635, 1542, 1417, 1058, and 1045 cm^−1^, the absorption peak at these points corresponds to N-H and O-H expansion vibration, unsaturated C-H stretching, C=O stretching, C=C deformation, bending vibration in saturated C=H plane, and unsaturated C-H plane bending vibration, respectively. In the UF, the absorption peaks at 3403, 1637, 1419, 1060 cm^−1^ move left compared to MF, while the absorption peaks at 3396,1540 cm^−1^ move right compared to the MF. The wide absorption peak of MF at 3398 cm^−1^ is lower than that of UF, indicating that more O-H bonds are broken.

#### 2.2.3. X-ray Diffraction Determination

X-ray diffraction (XRD) analysis revealed information about the crystal structure of the soybean residue. As shown in Figure 3b, all samples presented typical type I cellulose crystals, with the MF peak below the UF in all ranges, indicating that the mixed fermentation treatment reduced the crystallinity of the soybean residue samples; however, the peak position and width were not significantly different, indicating that the ordered structure of the cellulose crystallization region in MF was not disrupted. Low crystallinity can also directly affect the physical and chemical properties of dietary fiber such as water holding, oil holding, water expansion force, and cholesterol adsorption capacity.

#### 2.2.4. Physicochemical Properties

The results in Table 4 indicate that the mixed fermentation significantly (*p* < 0.001) increased the SDF yield. MF increased it by 167.15% compared to UF. Moreover, the increase in SDF content was accompanied by a decrease in IDF yield, which also confirms that the nature of DF modification is a conversion from IDF to SDF. The crude fat content, protein content, water content and ash content of mixed fermented dietary fiber are shown in Table 5.

The monosaccharide composition of both MF and UF is shown in Table 6. Both UF and MF contain nine monosaccharides, including mannose, rhamnose, glucuronic acid, galacturonic acid, glucose, galactose, xylose, arabinose, and fucose. In MF, all eight monosaccharides were lower than in UF, in addition to xylose. The levels of mannose, rhamnose, galactose, arabinose, and fucose were higher in UF than in MF, suggesting that mixed fermentation can increase hemicellulose degradation.

#### 2.2.5. Effect of Mixed Fermentation Modification on the Thermal Stability of Soybean Residue

**Figure 4:**
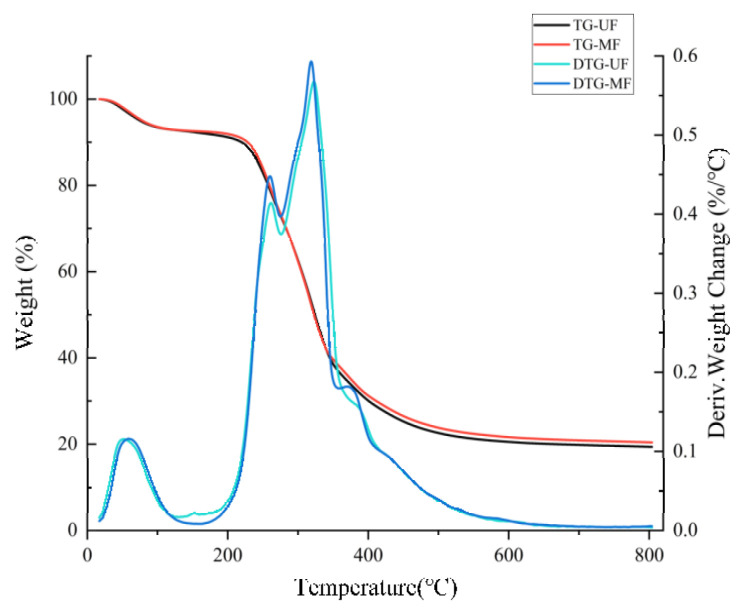
TGA-DSC analysis of unfermented and mixed fermented soybean residue. UF, unfermented soybean residue; MF, mixed fermented soybean residue.

It is well known that thermogravimetric analysis (TGA) and differential scanning calorimetry (DSC) analysis are widely used to test and study the thermal stability of soybean residue samples [15]. Figure 4 shows the three stages of heat decomposition of the soybean residue. In the first stage (30–200 °C), water was mainly lost, during which the sample mass decreased, and the weight of the two kinds of soybean residues changed relatively slowly. In the second stage, both the unmodified and modified soybean residues showed distinct and rapid weightlessness peaks between 200 and 400 °C, with the MF showing a more pointed shape compared to the UF. The third stage (400–600 °C), where the weight of the sample is slowly lost until equilibrium, is mainly a carbonization process.

### 2.3. Adsorption Capacity Analysis

#### 2.3.1. Oil Holding Capacity Determination

SDF is characterized by a high oil holding capacity (OHC), and plays an important role in physical health which includes hindering the absorption of lipids, promoting gastrointestinal peristalsis, and preventing obesity, etc. [16]. As shown in Table 4, the OHC of MF was significantly higher (*p* < 0.001) than that of UF, indicating that the mixed fermentation treatment greatly improved the retention capacity of the oil molecules. The increase in SDF content may also be the cause of the increase in OHC.

#### 2.3.2. Water Holding Capacity Determination

The water holding capacity (WHC) represents the ability of the soybean residues to retain moisture under external pressure [17]. The experimental results are shown in Table 4. Clearly, the WHC of the treated samples was significantly higher (*p* < 0.001) than that of the UF. In brief, MF had a stronger effect than UF on the loss of tissue water molecules, probably due to the fluffy, porous soybean residue caused by the mixed fermentation treatment.

#### 2.3.3. Water Swelling Capacity Determination

As shown in Table 4, the water swelling capacity (WSC) for MF was significantly higher (*p* < 0.001) than for UF. After fermentation, the WSC of the soybean residues increased by 75%. This result is consistent with the performance of the MF in both the OHC and the WHC.

#### 2.3.4. Cholesterol Adsorption Capacity Determination

**Figure 5:**
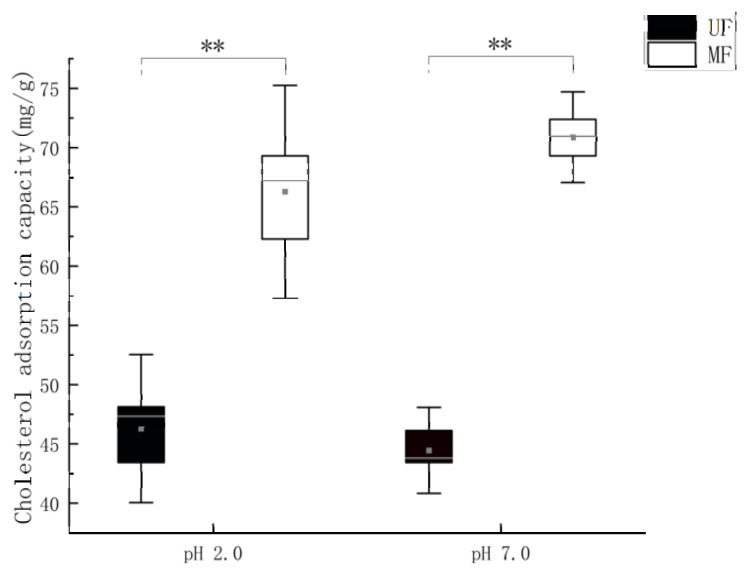
Cholesterol adsorption capacity of mixed fermented soybean residue. UF, unfermented soybean residue; MF, mixed fermented soybean residue. ** *p* < 0.01.

It is reported that SDF has hypolipidemic properties and can effectively mitigate the harm caused by cholesterol accumulation [18]. As shown in Figure 5, MF has a higher cholesterol adsorption capacity (CAC) in the simulated small intestinal environment (pH 7.0) when compared to the simulated gastric environment (pH 2.0). The CAC of MF was significantly higher (*p* < 0.01) than UF at both pH 2.0 and 7.0, probably due to fermentation changing the microstructure of the dietary fiber.

### 2.4. Analysis of Antioxidant Capacity

**Figure 6:**
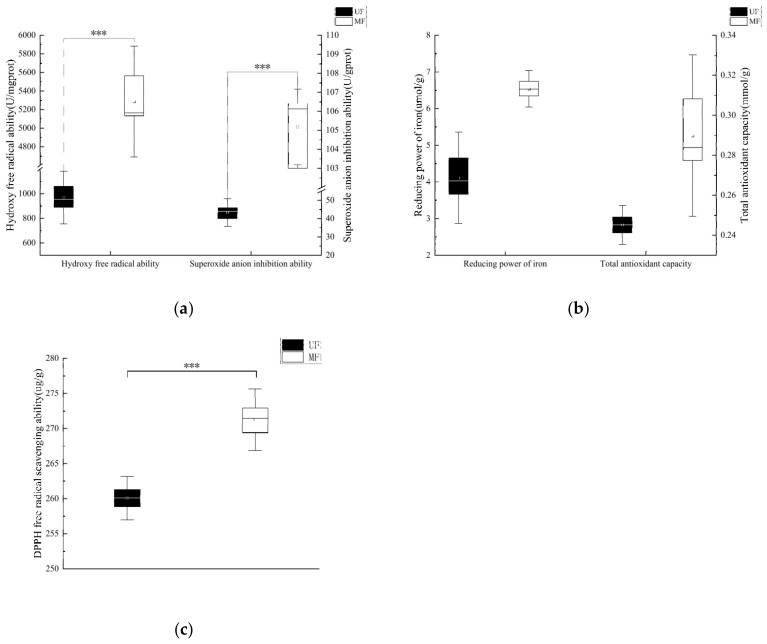
Analysis of the antioxidant capacity of mixed fermentation-modified soybean residue. (**a**) superoxide anion inhibition ability and hydroxy free radical ability; (**b**) reducing power of iron and total antioxidant capacity; (**c**) DPPH free radical scavenging ability. UF, unfermented soybean residue; MF, mixed fermented soybean residue. *** *p* < 0.001.

The superoxide anion inhibition ability, hydroxyl free radical ability, and DPPH free radical scavenging ability of MF and UF are shown in Figure 6a,c. The superoxide anion inhibition ability, hydroxy free radical ability, and DPPH free radical scavenging ability of MF were significantly higher (*p* < 0.001) than those of UF. This may be related to the fact that the mixed fermentation of LAB and SC destroyed the structure of soybean residue and promoted the decomposition of hemicellulose. The reducing power of iron and the total antioxidant capacity of MF and UF are shown in Figure 6b. The reducing power of iron and the total antioxidant capacity of MF were significantly higher than those of UF, but they were not statistically significant (*p* > 0.05).

## 3. Discussion

This study aimed to explore the optimal conditions for the mixed fermentation of LAB and SC and the effects of mixed fermentation on the structure, properties, and potential biological activity of soybean residue.

The SEM difference between UF and MF may be due to the lactic acid produced in the mixed fermentation process, which leads to a decrease in the pH of soybean residue, promotes the acidification of soybean residue, effectively damages its surface structure, leads to microstructure recombination, and improves the specific surface area of soybean residue [19].

The FT-IR analysis showed the characteristics of the main functional groups of the soybean residue, mainly showing the characteristic bands of cellulose, hemicellulose, and pectin. At the time of 3401 cm^−1^, the broad absorption peak is associated with stretching and bending vibrations of N-H and O-H groups, especially the intermolecular hydrogen bond interactions in cellulose and hemicellulose, with a lower peak strength in MF, indicating that cellulose and hemicellulose are resolved during fermentation [20]. Another absorption peak at 2927 cm^−1^ corresponds to the -CH_2_ (methylene) stretch vibration, belonging to the range of saturated C-H, which comes from the polypropylene group of polysaccharides [21]. For MF and UF, the absorption peak at 1542 cm^−1^ is correlated with cellulose [22]. The adsorption peak intensity of MF at 1417 cm^−1^ is higher than that of UF, which is mainly due to the disruption of the crystalline region of cellulose by LAB and SC during fermentation, resulting in an improvement in the peak strength; the absorption peak is related to pectin [23]. For UF and MF, there is a strong absorption peak at 1058 cm^−1^, possibly due to the tensile vibration of the C-O groups in xyloglucan [24,25]. The stretching vibration near 890 cm^−1^ may be caused by the β-glycosidic bond in DF, and the MF peak is slightly higher here than in UF, indicating that fermentation may disrupt the β-glycosidic bond of pectin and partial polysaccharides [26].

The XRD analysis revealed the structural and crystal information of the soybean residue, with all samples presenting typical type I cellulose crystals and a 13.3% reduction in the total crystallinity of MF compared to the UF group, indicating changes in the physicochemical properties of the cellulose fibers [27]. However, the crystal structures of the UF and MF are almost similar, indicating that fermentation does not change the type of crystal structure in the soybean residue. This is similar to the conclusions obtained from previous studies [28]. UF soybean residue has an obvious diffraction peak at 21.28° (2θ). After mixed fermentation, the diffraction peak shifted to 21.38° (2θ) and the peak strength decreased by 7.84%, indicating that mixed fermentation reduced the crystallinity of soybean residue, which may be due to the hydrolyzed cellulose and hemicellulose in soybean residue by LAB and SC during the fermentation process; this meant that the surface structure was eroded and broken, leading to the decrease in crystallinity. This is consistent with the results of the SEM. The reduced crystallinity at 15° (2θ) in the XRD pattern of the UF may be the result of the disruption of the original ordered cellulose structure [29].

The SDF content of the soybean residue after mixed fermentation modification was significantly increased (*p* < 0.001) by 167.15%, while the IDF content and TDF content decreased, but not significantly. This may be due to the degradation of wall polysaccharides and the destruction of cell walls caused by the fermentation of LAB and SC. IDF includes cellulose, hemicellulose and lignin, where hemicellulose includes glucose, xylose, mannose, arabinose and galactose, etc. Combined with monosaccharide composition analysis, it was found that compared with UF, the contents of glucose, mannose, arabinose and galactose in MF decreased obviously, which may be due to the degradation of IDF and the increase in SDF content [27,30]. Compared with UF, the xylose content in MF was higher. D-xylose can reduce postprandial blood glucose levels, inhibit adipogenesis and regulate lipid metabolism genes in obese mice, induced by a high-fat diet [31]. It also provides a feasible basis for MF to regulate the production and metabolism of fat. Rhamnose, galacturonic acid and fucose belong to pectin [32]. With UF, their content in MF decreased, indicating that the pectin content was not increased in the process of increasing the SDF content by mixed fermentation. From an industrial and production perspective, the mixed fermentation of LAB and SC has a significant effect on improving the SDF production, and helps to improve the added value of soybean residue. Mixed fermentation results in hemicellulose decomposition, which is consistent with the reduction in IDF yield described above.

The TGA-DSC combined analysis showed that MF has higher thermal stability than UF, probably due to the mixed fermentation modification promoting the decomposition of dietary fiber in the soybean residue. The weight loss of MF was slightly smaller than that of UF during thermal degradation, which is mainly reflected in the differences produced at 400–800 °C. The thermal stability of MF is increased, possibly because the fermentation modification increases SDF content. SDF is more thermostable than IDF, which is consistent with the results of previous studies [33,34].

The mixed fermentation treatment significantly (*p* < 0.001) improved the OHC, WHC and WSC of the soybean residue (Table 4). In the joint fermentation process of LAB and SC, IDF is decomposed, destroying the microstructure of soybean residue. The structure is looser, the adsorption capacity of water is increased, and more polar and non-polar groups are exposed. The mixed fermentation process significantly increased the content of SDF in soybean residue, and this increase in SDF content could improve the solubility. The process also enhanced the binding force of soybean residue with water and oil and improved the adsorption capacity of soybean residue. These results indicate that soybean residue modified by combined fermentation has excellent oil holding capacity, especially for saturated fat. High and excellent oil holding capacity is an important basis for the function of modified soybean residue, which can prevent the excessive absorption of fat in the intestine and provides a feasible basis for the improvement of intestinal health and intestinal peristalsis of soybean residue [35,36].

Mixed fermentation treatment significantly (*p* < 0.01) improved the cholesterol adsorption capacity of soybean residue in the gastrointestinal tract (Figure 5). Overall, the CAC of both UF and MF was higher at pH 7.0 than at pH 2.0, indicating that soybean residue had a stronger absorption capacity for cholesterol in the intestine than in the stomach, indicating that it could not only prevent excess cholesterol from entering the intestine, but also control the decomposition of cholesterol. The significant increase in CAC of MF over UF may be due to the destruction of the soybean residue structure. More pores and folds and increased SDF content may also be reasons. Meanwhile, DF may reduce cholesterol absorption by reducing micellar cholesterol solubility and inhibiting cholesterol absorption [37]. The relationship between cholesterol and gut bacteria is complicated. As a result, it is hypothesized that DF adsorption onto cholesterol by mixed fermentation modified soybean residue may aid in the maintenance of intestinal microbiological homeostasis, thereby indirectly maintaining intestinal health. This also provides a possibility for the regulation of cholesterol content by soybean dregs [38]. These results confirmed that the modified soybean residue produced by mixed fermentation had the effect of blocking the absorption of lipids and could be used as the raw material of lipid-reducing food. The antioxidant capacity of MF was significantly stronger than that of UF, and the superoxide anion inhibition ability, hydroxy free radical inhibition ability and DPPH radical scavenging ability were significantly improved (*p* < 0.001) (Figure 6). This may be related to the significantly increased SDF content in MF; moreover, the increased phenol content in the soybean residue after *Lactobacillus* fermentation may also be related [39].

## 4. Materials and Methods

### 4.1. Materials

Fresh soybean residue was provided by Kangle Tofu Workshop (Changchun, China). Soybean residue should be dried for 24 h at 55 °C before reuse. *Saccharomyces cerevisiae* strain BNCC337309 was obtained from the BeNa Culture Collection (Beijing, China). The KC205 LAB strain was isolated and screened from homemade pickled cabbage juice by the food microbiology team of the Agro-product Process Institute of the Jilin Academy of Agricultural Sciences, and has the characteristics of strong acid production and acid resistance.

High temperature-resistant α -amylase (2 × 10^4^ U/mL), papain (800 U/mg), and amyloglucosidase (1 × 10^5^ U/mL), were purchased from Shanghai Yuanye Bio-Technology Co., Ltd. (Shanghai, China). Peptone, yeast extract fermentation, glucose, NaCl, beef extract, anhydrous sodium acetate, sodium citrate, K_2_HPO_4_, MgSO_4_, MnSO_4_ and Tween 80 were all purchased from Sangon Biotech Co., Ltd. (Shanghai, China). All chemicals were of analytical grade or better.

### 4.2. Preparation of the Culture Medium

The LB liquid culture medium (g/L) was made up of 10.0 g of peptone, 3.0 g of yeast extract, 3.0 g of glucose and 5.0 g of NaCl, pH 6.2–6.4 and sterilized at 121 °C for 21 min.

The MRS liquid culture medium (g/L) was made up of 10.0 g peptone, yeast extract 5.0 g, beef extract 10.0 g, glucose 20.0 g, anhydrous sodium acetate 5.0 g, and sodium citrate 5.0 g, K_2_HPO_4_ 2.0 g. The pH value was 6.0–7.0, and then MgSO_4_ 0.2 g, MnSO_4_ 0.05 g, Tween 80 1.0 mL were added and fully dissolved. The medium was sterilized at 121 °C for 21 min.

The above LB liquid culture medium was used for cultivating SC and the above MRS liquid culture medium was used for cultivating LAB.

### 4.3. Preparation of Fermented Soybean Residue

#### 4.3.1. Strain Culture Method

SC seed solution preparation [11]: Under aseptic conditions, a single SC colony was added to sterilized LB liquid culture medium (200 mL) and incubated at 30 °C and 180 rpm for 48 h with constant temperature oscillation. The above cultured medium was added into the sterilized LB liquid culture medium (200 mL) at 5% inoculum volume, and incubated at 30 °C and 180 rpm for 48 h with constant temperature oscillation.

LAB seed solution preparation [40]: Under aseptic conditions, a LAB single colony was added to sterilized MRS liquid culture medium (200 mL) and incubated at 37 °C for 18 h. The above cultured medium was added into sterilized MRS liquid culture medium (200 mL) at 5% inoculating volume, and incubated at 37 °C for 18 h.

#### 4.3.2. Response Surface Methodology (RSM)

RSM was used to explore the relationship between the four controlled experimental variables (fermentation time, water content, inoculation proportion and inoculum amount) and the response value (SDF yield). After obtaining the appropriate range of these independent variables through univariate experiments, an RSM with four factors and three levels was generated by the Box–Behnken design (BBD).

#### 4.3.3. Mixed Fermentation

Under sterile conditions, SC seed solution and LAB seed solution were taken in different proportions. The effective viable counts of SC and LAB were 1~3 × 10^8^ cfu/mL and 1~2 × 10^8^ cfu/mL, respectively. The total volumes were 60 mL, 80 mL, 100 mL, respectively, and were centrifuged at 4000 rpm for 8 min. The volume of sterile normal saline needed was calculated, and the cells were redissolved based on the final water content of the soybean residue. They were added to soybean residue (100 g), labeled as 60%, 80% and 100% inoculation amount, respectively, and cultured at 37 °C.

### 4.4. Structural Analysis

#### 4.4.1. Scanning Electron Microscope (SEM) Analysis

The microstructures of the soybean residue samples were observed by SEM (Zeiss SUPRA5, Germany), slightly modified according to the method described by Li et al. [27]. Before testing and recording, soybean residue samples were attached to metal plates with conductive tape to facilitate sputtering gold plating. Each micrograph was taken at 1000× and 3000× magnification at 10 kV.

#### 4.4.2. Fourier Transform Infrared Spectroscopy Assay

The chemical composition changes of the soybean residue samples were analyzed by an FT-IR spectrometer (Nicolet 6700, Thermo Fisher Scientific, Waltham, MA, USA), slightly modified according to the method described by Lin et al. [41]. Soybean residue samples were mixed with KBr (1:100 *w*/*w*) for grinding and pressing. The mixture was immediately placed in the optical path for scanning, and FT-IR spectra from 400–4000 cm^−1^ with a resolution of 4 cm^−1^ were recorded.

#### 4.4.3. Crystallinity Determination

The method of crystallinity determination was slightly modified from Ma et al. [42]. The XRD curves of the soybean residue samples were obtained using an X-diffractometer (D/Max-Ultima +, Rigaku Corporation, Tokyo, Japanese) with the following parameters: an operating voltage and current of 30 kV and 20 mA, respectively, with a range of 5–90° and a step size of 0.02°. The X-ray source was Cu, and relative crystallinity was calculated by Jade 6.5.

#### 4.4.4. Determination of Basic Physicochemical Composition of DF from Soybean Residue

Protein content was measured by the Kjeldahl method, crude fat content was determined by the Soxhlet extraction method, water content was determined by the drying method, and ash content was determined by the incineration method [43]. SDF extraction and determination in all samples were performed as described by Chu et al. [20]. In addition, the extraction and determination of IDF and TDF were measured in accordance with the National Food Safety Standard (GB/T 5009.88-2014) of China [44].

The composition of monosaccharides in soybean residue was analyzed by liquid chromatography (Agilent 1200, Santa Clara, USA); the mobile phase A was 0.1 mol/L 0.1 M KH_2_PO_4_ (pH 6.8), the flow phase B is acetonitrile, and the flow phase gradient is A: B = 82: 18, equal degree elution; the flow rate is 1.0 mL/min and the sample size is 10 μL [45,46,47]. The composition of single sugars is calculated as follows:

W = (C − C_0_) × V × N/m (2)

where W represents the monosaccharide content, mg/kg; C represents the monosaccharide concentration, mg/L; C_0_ represents the monosaccharide concentrations in the blank control, mg/L; V represents the constant volume, mL; N represents the dilution multiple; and m represents the sampling amount of the sample, g.

#### 4.4.5. Thermal Performance

Thermal weight analysis SDT Q600 (TA Instruments Inc., New Castle, USA) was used to test the thermal performance of soybean residue samples. Referring to the previous methods, with slight modifications [41], different soybean residue samples were placed in a nitrogen atmosphere (20 mL/min). and by SDT Q600 test were heated from 30 °C to 600 °C at a heating rate of 20 °C/min.

### 4.5. Functional Features

#### 4.5.1. Oil Holding Capacity

Soybean residue samples (1.0 g) were maintained with 20 mL of vegetable oil at 26 °C for 24 h, and after centrifugation at 4500 r/min for 12 min at room temperature, the precipitate was collected [14]. The OHC (g/g) is represented by the following formula:

OHC(g/g) = (M_2_ − M_1_)/M_1_ (3)

where M_2_ is the wet weight of the sediment, the M_1_ is the weight of the dried soybean residue sample.

#### 4.5.2. Water Holding Capacity

After holding the soybean residue samples (1.0 g) with 20 mL of ultrapure water at 26 °C for 24 h and centrifuging at 4500 rpm for 12 min at room temperature, the supernatant was discarded and the precipitate was collected [14]. The WHC is calculated as follows:

WHC(g/g) = (W_1_ − W_0_)/W_0_ (4)

among which W_1_ is the weight of the sample after centrifugation and W_0_ is the weight of the sample before centrifugation.

#### 4.5.3. Water Swelling Capacity

The water swelling capacity of soybean residue samples was determined according to previous methods and slightly modified [27,48]. The soybean residue sample (0.5 g) was mixed with distilled water (10 mL) in a 37 °C scale tube for 24 h, and the sample volumes of the sample before and after expansion were read. The WSC is calculated according to the following formula:

WSC (mL/g) = (V_1_ − V_2_)/M_1_ (5)

among which V_1_ is the expanded volume, V_2_ is the volume before the expansion, and M_1_ is the dry sample weight.

#### 4.5.4. Cholesterol Adsorption Capacity

The CAC of soybean residue samples was adjusted slightly according to the method of Yang et al. [29]. In brief, the yolk and distilled water were mixed in a ratio of 1:9. The soybean residue sample (0.1 g) was mixed with emulsion (5 mL) in a conical flask, then adjusted to pH 2.0 (simulated gastric environment) or 7.0 (simulated small intestine) and oscillated at 37 °C for 2 h. After centrifugation at 4500 rpm for 12 min, the supernatant was collected and diluted with acetic acid (1:10 *v*/*v*) to determine the cholesterol content in the supernatant. The cholesterol content of the different treatment groups was determined by the o-phthalaldehyde method. The CAC is calculated as follows:

CAC (mg/g) = (W_2_ ࢤ W_1_)/M_1_ (6)

among which W_2_ is the amount of emulsified cholesterol, W_1_ is the adsorbed cholesterol content, and M_1_ is the weight of the dried soybean residues.

### 4.6. Determination of Antioxidant Capacity

The superoxide anion inhibition ability, hydroxy free radical inhibition ability, DPPH free radical scavenging activity, and total antioxidant capacity were determined according to the method described by the kit manufacturer (Nanjing Jiancheng Bioengineering Institute, Nanjing, China) (Supplementary Materials). The capacity of iron ion reduction was determined according to the method described by the kit manufacturer (Suzhou Grace Biotechnology Co., Ltd., Suzhou, China) (Supplementary Materials [49,50]).

### 4.7. Statistical Analysis

Experimental results are expressed as the mean ± standard deviation (*n* ≥ 3), and the response surface design was performed using Design Expect 11.0.4. Statistical analysis and chart mapping were completed using IBM SPSS Statistics 21.0 and Origin 9.0 with a significance level of *p* < 0.05.

## 5. Conclusions

In this study, we selected two strains, SC and LAB, for mixed fermentation of soybean residue. The conditions of mixed fermentation modified soybean residue were optimized. The optimal water content is 77.22%; the optimal inoculation proportion (L:S) is 6:5; the optimal inoculation amount is 81.67%; the optimal fermentation time is 72.32 h, and after fermentation, the SDF content is 3.62 g/100 g. Meanwhile, the structural features and physicochemical properties were analyzed. After fermentation, the soybean residue has a typical absorption peak of polysaccharides and a typical crystal structure of type I cellulose. Dietary fiber showed more pores and loose structures with higher thermal stability. Soybean residue after mixed fermentation has good adsorption properties, both physical and chemical, and high antioxidant activity in vitro, with potential applications in food processing, functional food production or adjuvant treatment of some diseases. In conclusion, mixed fermentation is an effective way to improve the processing performance and functional activity of soybean residue, which provides a new idea for improving the utilization rate of soybean residue.

## Figures and Tables

**Table 1:** Factors and levels in the response surface methodology.

Levels	Water Content (%)	Inoculation Proportion (L:S)	Inoculation Amount (%)	Time (h)
−1	70	1:2	60	48
0	80	1:1	80	72
1	90	2:1	100	96

**Table 2:** Experimental data of the four-factor, three-level Box–Behnken designed for RSM and study response.

Runs	Water Content (%)	Inoculation Proportion (L:S)	Inoculation Amount (%)	Time(h)	Soluble Dietary Fiber Content (g/100 g)
1	90	1:1	60	72	2.8695
2	80	1:1	80	72	3.6770
3	80	1:1	80	72	3.6305
4	80	1:1	80	72	3.5278
5	70	2:1	80	72	3.0139
6	80	1:1	60	96	2.5454
7	80	2:1	100	72	3.0155
8	80	2:1	80	48	2.4095
9	80	1:1	100	96	2.8960
10	70	1:1	60	72	3.2147
11	80	1:1	60	48	2.8762
12	90	1:1	80	96	2.4063
13	70	1:1	80	96	2.7680
14	70	1:1	80	48	2.7270
15	80	1:1	100	48	2.5941
16	80	1:2	100	72	3.1628
17	70	1:2	80	72	3.1569
18	80	1:1	80	72	3.4516
19	80	1:2	80	48	2.6605
20	90	1:1	100	72	2.8524
21	90	1:1	80	48	2.3874
22	90	1:2	80	72	2.7265
23	70	1:1	100	72	3.2867
24	80	2:1	80	96	2.6494
25	80	2:1	60	72	3.0354
26	90	2:1	80	72	2.7045
27	80	1:1	80	72	3.6325
28	80	1:2	60	72	3.0258
29	80	1:2	80	96	2.5094

**Table 3:** ANOVA with the response face quadratic model.

Source	Sum of Squares	Df	Mean Square	F value	*p* Value	Significance ^a^
Model	4.08	14	0.29	107.87	<0.0001	***
A -Water content	0.41	1	0.41	152.18	<0.0001	***
B -Inoculation proportion	0.014	1	0.014	5.28	0.0375	*
C -Inoculation amount	0.000482	1	0.00482	1.79	0.2028	
D -Time	0.001196	1	0.001196	0.44	0.5165	
AB	0.00366	1	0.00366	1.36	0.2638	
AC	0.001985	1	0.001985	0.74	0.4057	
AD	0.0001221	1	0.0001221	0.045	0.8347	
BC	0.006154	1	0.006154	2.28	0.10534	
BD	0.038	1	0.038	14.15	0.0021	**
CD	0.1	1	0.1	37.06	<0.0001	***
A^2^	0.75	1	0.75	277.62	<0.0001	***
B^2^	0.77	1	0.77	286.61	<0.0001	***
C^2^	0.22	1	0.22	79.89	<0.0001	***
D^2^	2.96	1	2.96	1096.21	<0.0001	***
Residual	0.038	14	0.0027			
Lack of fit	0.003952	10	0.003952	0.047	0.9999	Not significant
Pure error	0.034	4	0.008463			
Cor total	4.12	28				
R-squared	0.9908					
Adj. R-squared	0.9816					
Adeq. precision	32.243					
C.V.%	1.76					

Significance ^a^: * *p* < 0.05; ** *p* < 0.01; *** *p* < 0.001.

**Table 4:** Functional characteristics of UF and MF.

Sample	SDF (g/100 g)	IDF (g/100 g)	TDF (g/100 g)	OHC (g/g)	WHC (g/g)	WSC (g/g)
UF	1.37 ± 0.28	88.15 ± 4.15	89.52 ± 5.14	2.466 ± 0.34	11.336 ± 0.63	4.0 ± 0.5
MF	3.62 ± 0.03 ***	84.96 ± 5.24	88.67 ± 4.19	10.699 ± 0.53 ***	16.856 ± 0.97 ***	7.0 ± 0.25 ***

Significance: *** *p* < 0.001. The data are expressed as mean ± standard deviations (*n* = 3). UF, unfermented soybean residue; MF, mixed fermented soybean residue; SDF, soluble dietary fiber; IDF, insoluble dietary fiber; TDF, total dietary fiber; OHC, oil holding capacity; WHC, water holding capacity; WSC, water swelling capacity.

**Table 5:** Physicochemical properties of unfermented and mixed fermented soybean residue.

Sample	Crude Fat (%)	Protein (g/100 g)	Water (%)	Ash content (%)
UF	6.7 ± 1.2	16.2 ± 2.5	7.8 ± 1.6	4.08 ± 0.71
MF	6.9 ± 0.9	16.9 ± 2.1	4.9 ± 0.3 *	3.31 ± 1.08

The data are expressed as mean ± standard deviations (*n* = 3). UF, unfermented soybean residue; MF, mixed fermented soybean residue. * *p* < 0.05.

**Table 6:** Monosaccharide composition of unfermented and mixed fermented soybean residue.

Name	UF (g/kg)	MF (g/kg)
Mannose	17.011 ± 4.329	10.397 ± 0.981
Rhamnose	25.355 ± 7.457	22.953 ± 5.064
Glucuronic acid	3.143 ± 1.047	2.844 ± 0.976
Galacturonic acid	33.178 ± 3.115	30.483 ± 4.853
Glucose	18.959 ± 5.752	17.589 ± 2.611
Galactose	168.372 ± 31.623	157.096 ± 27.352
Xylose	44.563 ± 8.391	45.911 ± 9.407
Arabinose	93.193 ± 14.067	81.567 ± 9.225
Fucose	16.663 ± 5.584	14.538 ± 3.428

The data are expressed as mean ± standard deviations (*n* = 3). UF, unfermented soybean residue; MF, mixed fermented soybean residue.

## Data Availability

Not applicable.

## Supplementary Materials

The following supporting information can be downloaded at: https://www.mdpi.com/article/10.3390/molecules28031322/s1.

## Sample Availability

Samples of the compounds are available from the authors.
