# Supplementary material for: Optimization of Mixed Fermentation Conditions of Dietary Fiber from Soybean Residue and the Effect on Structure, Properties and Potential Biological Activity of Dietary Fiber from Soybean Residue
Source: Molecules. 2023 Jan 30;28(3):1322. doi: 10.3390/molecules28031322 (PMC9920189; doi:10.3390/molecules28031322)
Supplement: Supplementary file 1 [file molecules-28-01322-s001.zip › molecules-2096796-supplementary V2.pdf]

Comparisons of optimization of mixed strain fermentation conditions of dietary fiber from soybean residue and the effect on structure, properties and potential biological activity of dietary fiber from soybean residue

Supplementary methods

Determination of antioxidant capacity

Determination of inhibition ability of superoxide anion. Simply put, 0.15 mg/mL of vitamin C solution (0.05 mL), water (0.05 mL) and sample (0.05 mL) were mixed with superoxide anion solution (1.3 mL), respectively, and then in a constant temperature water bath of 37 °C for 40 min. After adding color developing agent (2 mL), the mixture was mixed and left for 10 min at room temperature. The absorbance was read at 550 nm. The calculation formula is as follows:

$$\text{Superoxide anion inhibition ability (U/gprot)}=(A_{control}-A_{sample})/(A_{control}-A_{standard})*150/Cpr \quad (7)$$

among A_{sample} represents the absorbance of the sample; $A_{control}$ represents the absorbance of water; $A_{standard}$ represents the absorbance of 0.15 mg/mL vitamin C solution; Cpr represents the protein concentration of the sample.

Determination of inhibition ability of hydroxy free radical [49]. To put it simply, 0.03% H_2O_2 (0.2 mL), water (0.4 mL), water (0.2 mL) and sample (0.2 mL) were mixed with hydroxyl radical solution (0.4 mL), respectively, at 37 °C, reaction for 1 min, immediately added color developing agent (2 mL) and mixed, at room temperature for 20 min. The absorbance was read at 550 nm. The calculation formula is as follows:

$$\text{Hydroxyl free radical inhibition ability (U/mgprot)}=(A_{control}-A_{sample})/(A_{standard}-A_{black})*44.12/Cpr \quad (8)$$

among A_{sample} represents the absorbance of the sample; $A_{control}$ represents the absorbance of water (0.2 mL); $A_{standard}$ represents the absorbance of 0.03% H_2O_2; A_{black} represents the absorbance of water (0.4 mL); Cpr represents the protein concentration of the sample.

Determination of DPPH free radical scavenging ability [50]. In simple terms, the sample (0.1 g) was mixed with 80% methanol solution (1 mL), homogenized in ice water bath, centrifuged at 12000 rpm for 10 min, and the supernatant was collected. The supernatant (400 μL) was mixed with DPPH radical solution (600 μL). The supernatant (400 μL) was taken from each sample and mixed with 80% methanol (600 μL). The 80% methanol (400 μL) was mixed with DPPH radical solution (600 μL). Room temperature dark for 30 min. Centrifuge at 4000 rpm for 5 min and collect supernatant. The absorbance was read at 517 nm. The calculation formula is as follows:

$$\text{DPPH free radical scavenging activity (μg/g)} =((1-(A_{supernatant}-A_{control})/A_{black})-0.0094)/0.032 \quad (9)$$

among $A_{supernatant}$ represents the absorbance of the sample after mixing with DPPH radical solution; $A_{control}$ represents the absorbance of the sample after mixing with 80% methanol; A_{black} represents the absorbance of 80% methanol after mixing treatment with DPPH radical solution.

Determination of total antioxidant capacity. Simply put, the sample (0.01 mL) was mixed with ABTS solution (0.19 mL) at room temperature for 6 min. The absorbance was read at 405 nm. The calculation formula is as follows:

$$\text{Total antioxidant capacity (mmol/g)} = (0.1524 \times A_{sample} + 0.4451)/Cpr \quad (10)$$

among A_{sample} represents the absorbance of the sample; Cpr represents the protein concentration of the sample.

Determination of reducing power of iron ions [50]. In simple terms, the sample (0.1g) was mixed with 80% ethanol solution (1 mL) and homogenized. Then, the supernatant was extracted by ultrasonic extraction for 30 min at 60 °C and 200-300 W, and centrifuged at 12000 rpm for 10 min. Supernatant (0.005 mL), water (0.025 mL) and color developing solution (0.17 mL) were blended. At the same time, water (0.03 mL) and color developing solution (0.17 mL) were mixed. At room temperature for 10 min, the absorbance was read at 590 nm. The calculation formula is as follows:

$$\text{Reducing power of iron } (\mu mol/g) = 3.12 \times (A_{sample} - A_{black} + 0.0165)/Cpr \quad (11)$$

among A_{sample} represents the absorbance of the sample; A_{black} represents the absorbance of water; Cpr represents the protein concentration of the sample.

References

49. Tang, C., Wang, Y., Long, Y., An, X., Shen, J., Ni, Y. Anchoring 20 (R)-ginsenoside Rg3 onto cellulose nanocrystals to increase the hydroxyl radical scavenging activity. *ACS SUSTAIN CHEM ENG*. **2017,** *5*(9), 7507-7513. https://doi.org/10.1021/acssuschemeng.6b02996

50. Cong-Cong, Q., Fan-Kun, Z., Na-Na, W., Bin, T. Functional, physicochemical and structural properties of soluble dietary fiber from rice bran with extrusion cooking treatment. *Food Hydrocolloids*. **2021,** 121, 107057. https://doi.org/10.1016/j.foodhyd.2021.107057
